# Treatment outcome and toxicity of intensity-modulated (chemo) radiotherapy in stage III non-small cell lung cancer patients

**DOI:** 10.1186/1748-717X-7-150

**Published:** 2012-09-07

**Authors:** Stephanie LA Govaert, Esther GC Troost, Olga CJ Schuurbiers, Lioe-Fee de Geus-Oei, Ariën Termeer, Paul N Span, Johan Bussink

**Affiliations:** 1 Department of Radiation Oncology, Radboud University Nijmegen Medical Centre, PO Box 9101, Nijmegen 6500 HB, The Netherlands; 2 Department of Pulmonary Diseases, Radboud University Nijmegen Medical Centre, PO Box 9101, Nijmegen 6500 HB, The Netherlands; 3 Department of Nuclear Medicine, Radboud University Nijmegen Medical Centre, PO Box 9101, Nijmegen 6500 HB, The Netherlands; 4 Department of Pulmonary Diseases, Canisius-Wilhelmina Hospital, Nijmegen The Netherlands

**Keywords:** Intensity-modulated (chemo)radiotherapy, Stage III non-small cell lung cancer

## Abstract

**Purpose:**

The aim of this retrospective cohort study was to assess treatment outcome, and acute pulmonary and esophageal toxicity using intensity modulated (sequential/concurrent chemo)radiotherapy (IMRT) in locally advanced stage III non-small cell lung cancer (NSCLC).

**Methods and materials:**

Eighty-six patients with advanced stage NSCLC, treated with either IMRT only (66 Gy) or combined with (sequential or concurrent) chemotherapy were retrospectively included in this study. Overall survival and metastasis-free survival were assessed as well as acute pulmonary and esophageal toxicity using the RTOG Acute Radiation Morbidity Scoring Criteria.

**Results:**

Irrespective of the treatment modality, the overall survival rate for patients receiving 66 Gy was 71% (±11%; 95% CI) after one year and 56% (±14%) after two years resulting in a median overall survival of 29.7 months. Metastasis-free survival was 73% (±11%) after both one and two years. There were no statistically significant differences between the treatment groups. Treatment related esophageal toxicity was significantly more pronounced in the concurrent chemoradiotherapy group (*p* = 0.013) with no differences in pulmonary toxicity.

**Conclusions:**

This retrospective cohort study in advanced non-small cell lung cancer patients shows that IMRT is an effective technique with acceptable acute toxicity, also when (sequentially or concomitantly) combined with chemotherapy.

## Introduction

Lung cancer is an increasing cause of death in developing and developed countries, accounting for 1.39 million deaths worldwide in 2008. Non-small cell lung cancer (NSCLC) accounts for approximately 85% of all lung cancer cases [1]. The treatment of choice for individuals with local NSCLC, stage I, II and IIIA (T3N1), is surgical resection. Based on large series of resected stage I and II NSCLC, five-year survival rates are commonly reported to be 60 to 80% and 40 to 50%, respectively [2]. In patients with locally advanced NSCLC, stage IIIA (N2) and IIIB, concurrent radiochemotherapy is recommended [3,4]. However, survival rates with these approaches are only in the order of 15%, and a lot of patients are not eligible to undergo this intensified regimen [5,6]. Considering the fact that treatment failure most often occurs at the primary tumor site, improvement in overall survival of locally advanced stage III NSCLC can be achieved through better local control. A meta-analysis by Aupérin *et al*. proved concurrent regimens to be superior to sequential ones in terms of locoregional control and overall survival [5]. In concurrent schedules, the chemotherapeutic agents enhance the tumor’s radiosensitivity and thus the local treatment efficacy, but this comes at the cost of increased toxicity.

Apart from the concurrent administration of chemotherapy, another approach to accomplish better locoregional control is dose escalation. Several studies have shown that this strategy improves local control and consequently overall survival of locally advanced stage III NSCLC [7-11]. Using three-dimensional conformal radiation therapy (3D-CRT) allows dose escalation without excessive toxicity, while improving overall survival rates [12,13]. Compared with 3D-CRT, intensity-modulated radiation therapy (IMRT) enables even tighter sculpting of high-dose regions around the tumor volume, creates steep dose gradients and thus reduces radiation dose to surrounding normal tissues, ultimately facilitating dose-escalation [14]. Therefore, the University of Texas M.D. Anderson Cancer Center investigated the rate of high-grade treatment-related pneumonitis in patients with advanced NSCLC treated with concurrent chemotherapy and IMRT. Toxicity rates were compared with a similar cohort of patients treated with 3D-CRT (median radiation dose 63 Gy for both treatment modalities). The levels of Grade ≥3 radiation pneumonitis at 12 months according to RTOG toxicity scoring [15] were significantly (*p =* 0.002) lower for IMRT than for 3D-CRT, being 8% (95% CI 4%–19%) and 32% (95% CI 26%–40%), respectively [16]. This initial evaluation is consistent with the conclusion of a subsequent study of the institution in larger patient groups and with longer follow-up times [17]. Of the 496 NSCLC patients, 318 were treated with CT/3D-CRT and 91 with 4DCT/IMRT. The hazard ratio for 4DCT/IMRT was 0.33 (95% CI 0.13-0.82; *p =* 0.017) for Grade ≥3 radiation pneumonitis, indicating lower toxicity rates were associated with 4DCT/IMRT. These findings were confirmed by other studies [14,18]. Furthermore, IMRT reduces radiation doses to the esophagus, heart and spinal cord [18,19].

Published clinical data on outcome and toxicity using IMRT in stage III NSCLC are scarce. Therefore, the aim of the present retrospective cohort study was to evaluate outcome, and acute pulmonary and esophageal toxicity using intensity modulated (sequential/concurrent chemo)radiotherapy in locally advanced stage III NSCLC.

## Methods and materials

### Patient characteristics

All patients with advanced-stage irresectable NSCLC treated with curative intent at our institution between March 2008 and February 2011, eighty-six in total, were retrospectively included in this study. All research was carried out in compliance with the Helsinki Declaration and in accordance with Dutch law. The Institutional Review Board waved review due to the retrospective nature of this study. All primary tumors and the mediastinal N2 disease were cytologically or histologically proven. Prior to treatment initiation, patients underwent total body ^18^F-fluorodeoxyglucose positron emission tomography combined with low dose computed tomography (FDG PET-CT) and MRI scan of the brain for tumor staging purposes. Patients in good general condition were treated with concurrent chemoradiotherapy, those with a contraindication for chemotherapy were treated by radiation alone, and all remaining patients were treated with a sequential chemotherapy and radiotherapy. The planned radiation dose to the primary tumor and metastatic mediastinal lymph nodes using IMRT was 66 Gy in 33 fractions delivered five times per week. Chemotherapeutic agents in the sequential regimen typically consisted of three courses of gemcitabine (1250 mg/m^2^; on day 1 and 8) and cisplatinum (80 mg/m^2^; on day 1). The concurrent schedules varied between referring hospitals; in Radboud University Nijmegen Medical Centre it consisted of two courses of etoposide (100 mg/m^2^; on day 1–3) and cisplatinum (50 mg/m^2^; on day 1 and 8), in Canisius-Wilhelmina Hospital one course of gemcitabine/cisplatinum was administered prior to irradiation and two courses of etoposide/cisplatinum concurrently with radiation therapy.

### Organ segmentation and treatment planning technique

The gross tumor volume (GTV) encompassed the primary tumor volume defined on a contrast-enhanced slow-CT scan and the positive mediastinal lymph nodes as defined on CT imaging (short axis > 1 cm or necrosis). Besides, it included cytologically or histopathologically confirmed pathological FDG-avid lesions. Subsequently, the planning target volume (PTV) was created following the institute’s guidelines. Automatic contouring of the lungs and heart was performed using the Pinnacle^3^ treatment planning system (version 8.0 h; Philips Radiation Oncology Systems, Fitchburg, USA). Manual correction was performed if necessary. The esophagus was delineated from the lower border of the cricoid cartilage to the gastro-esophageal junction. The spinal cord was considered to be at the inner margin of the entire bony thoracic spinal canal.

An IMRT treatment plan was generated using a 3D convolution/superposition method for dose calculation, and a standard radiation beam geometry not encompassing the healthy contralateral lung. Multi-segment fields were generated for IMRT delivery on a step-and-shoot linear accelerator (Elekta SLi; Elekta AB, Stockholm, Sweden) using six co-planar 10 MV photon beams. Plans had been limited to 60 segments with a minimum segment area of 6 cm^2^ and at least 10 monitor units. Treatment plans had been optimized using an in-house developed class solution for inverse treatment planning with the direct machine parameter optimization algorithm producing deliverable beam segments. All plans had been normalized to a mean dose of 66 Gy in 33 fractions and satisfied the −5% and +7% dose heterogeneity criteria for the PTV according to ICRU 50/62 guidelines [20,21]. Routinely, position verification was performed using EPID or, from 2009 onwards, MV conebeam-CT imaging with an offline (NAL; no action level [22]) protocol (fraction 1, 2, 3 and weekly thereafter).

### Assessment of pulmonary and esophageal toxicity

During the course of radiation delivery, patient toxicity was weekly assessed by the treating physician according to the RTOG acute toxicity scoring criteria [15]. Pulmonary and esophageal toxicities were scored on a 6-point scale. For pulmonary toxicity grade 0 was defined as absence of symptoms, grade 1 as mild dry cough or dyspnea on exertion, grade 2 as persistent cough requiring narcotic antitussive agents, grade 3 as severe cough unresponsive to narcotic antitussive agent requiring steroids and intermittent oxygen, and grade 4 as severe respiratory insufficiency requiring continuous oxygen or assisted ventilation. For esophageal toxicity, grade 0 was defined as absence of symptoms, grade 1 as slight symptoms requiring no or non-narcotic analgesics, grade 2 as symptoms requiring narcotic analgesics and adapted diet, grade 3 as severe dysphagia with dehydration or weight loss requiring nasogastric tube feeding of intravenous fluids, and grade 4 complete obstruction, ulceration, perforation or fistula. For both toxicities grade 5 was considered toxicity related death.

### Statistical analysis

We retrospectively reviewed the records of the patients in the cohort until March 31, 2011. Clinical end points were overall survival and metastasis-free survival. Metastasis-free survival was based on clinical basis and, if metastases were suspected, adequate imaging was performed, *e.g.*, liver ultrasound, CT of thorax/abdomen, FDG-PET or bone-scan. These clinical end points with respect to the different treatment groups were calculated using the Kaplan-Meier method (GraphPad Prism, version 4.0, GraphPad Software, La Jolla, USA). Due to the retrospective nature of this study including patients from several referring hospitals, no reliable data on local and regional control were available and thus not analyzed. For comparison of pulmonary and esophageal toxicity between the different treatment schedules, the chi-square test was used. A *p*-value below 0.05 indicated statistical significance and was assessed using Log Rank test.

## Results

### Patients

The characteristics of the 86 patients are presented in Table1. Median age was 67 years, ranging from 45 to 83 years. Three patients with stage IIB (of whom one was treated for a recurrent disease), 53 with stage IIIA and 30 patients with stage IIIB were included in the study. Seven patients were treated with radiotherapy alone, 42 with sequential chemoradiotherapy, and 37 with concurrent chemoradiotherapy. Three of the 42 patients who received sequential chemotherapy and radiotherapy did not complete the planned three courses of chemotherapy due to malaise, a gastrointestinal bleeding and an exacerbation of pancreatitis. Eighty-one percent of the patients was treated with a radiation prescription dose of 66 Gy, one patient received 67.5 Gy in 2.25 Gy fractions (stage IIB patient) and 15 received less than 66 Gy due to clinical deterioration or the development of metastatic disease during the course of treatment. As a consequence the median irradiated dose was 66 Gy with a range from 32 to 67.5 Gy. Median follow-up for all patients was 12 months (range, 1–35 months), and for patients still alive at study closure date it was 17 months (range, 5–39 months).

**Table 1:** **Patient characteristics (n = 86); TNM classification according to 7**^**th **^**edition of International Association for the Study of Lung Cancer**

*Age (y)*	
median [range]	67.2 [44.6; 82.7]
*Morphology* (n)	
squamous cell carcinoma	33
adenocarcinoma	34
neuroendocrine carcinoma	1
non-small cell carcinoma	3
*Stage* (n)	
IIB	3
IIIA	53
IIIB	30
*T-stage* (n)	
T0	2
T1	16
T2	30
T3	18
T4	20
*N-stage* (n)	
N0	8
N1	4
N2	59
N3	14
unknown	1
*Treatment* (n)	
concurrent RT and CHT	37
sequential RT en CHT	42
RT alone	7

n = number of patients, y = years, RT = radiotherapy; CHT = chemotherapy.

### Treatment outcome

**Figure 1:**
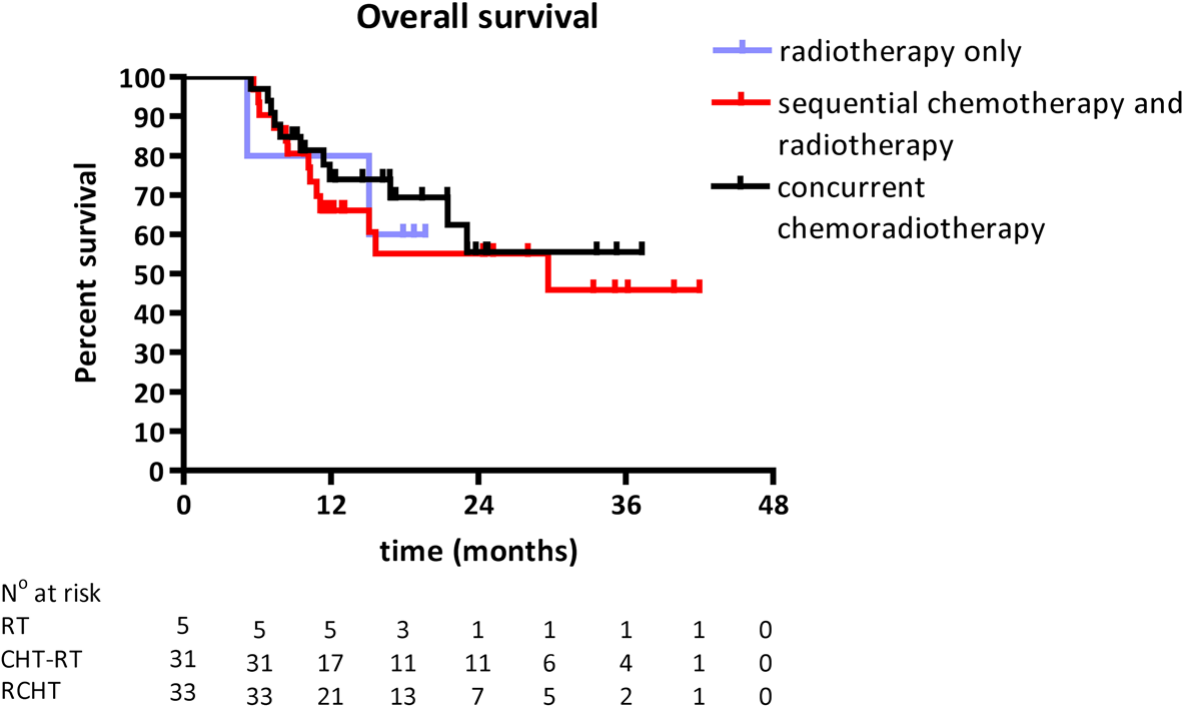
Kaplan-Meier estimate of overall survival in patients receiving a radiation dose of 66 Gy only, or combined with sequential or concurrent chemotherapy.

The median overall survival among the 70 patients receiving a radiation dose of 66 Gy was 29.7 months (Figure 1). The follow-up of patients treated with radiotherapy alone and concurrent chemoradiotherapy was too short to enable the calculation of the median overall survival time. In patients treated with sequential chemotherapy and radiotherapy the median overall survival was 29.7 months. Irrespective of the treatment modality, this resulted in an overall survival rate of 71% (±11%; 95% CI) after one year and 56% (±14%) after two years for patients receiving 66 Gy. In patients treated with radiation only, 1- and 2-year overall survival rates were 80% (55-100%) and 60% (18-100%), respectively. In patients treated by sequential chemotherapy and radiotherapy 1- and 2-year overall survival rates were 66% (±17%) and 55% (±20%), and in patients treated with concurrent chemoradiotherapy 74% (±16%) and 56% (±22%), respectively. No statistically significant differences in overall survival between the treatment groups were observed.

**Figure 2:**
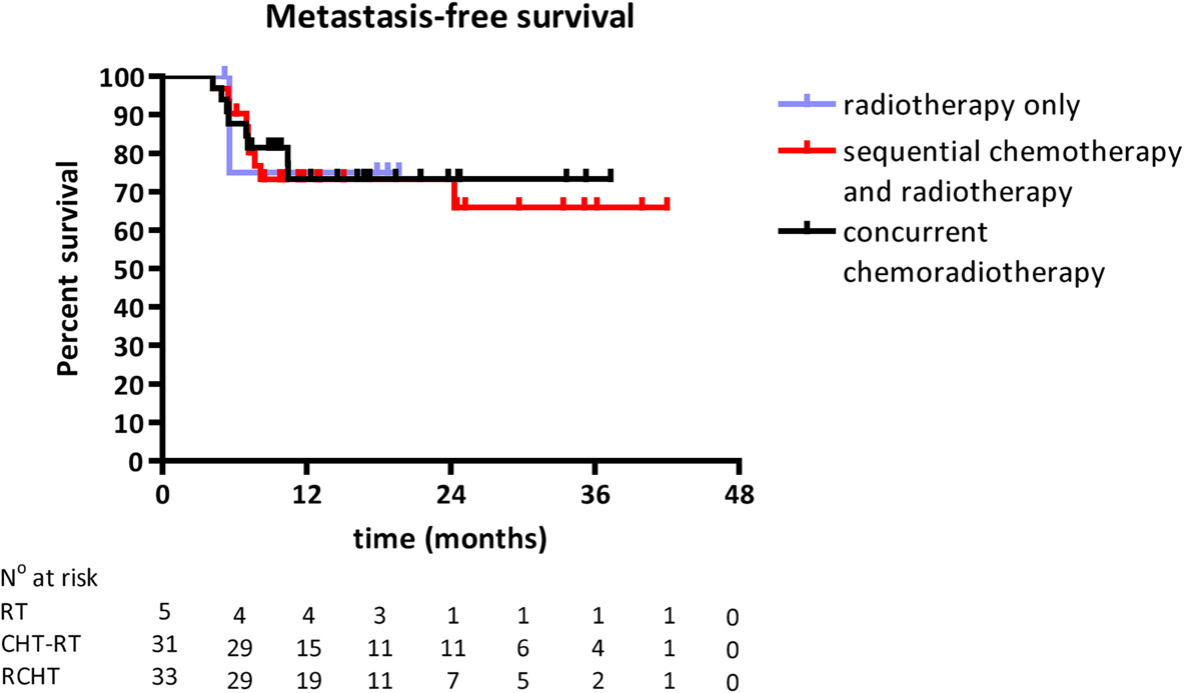
Kaplan-Meier estimate of metastasis-free survival in patients receiving a radiation dose of 66 Gy only, or combined with sequential or concurrent chemotherapy.

The median metastasis-free survival of patients receiving a radiation dose of 66 Gy was not reached in any of the treatment schedules, and after 1 and 2 years, the metastasis-free survival rates were both 73% (±13%) (Figure 2). Among patients receiving a radiation dose of 66 Gy without additional chemotherapy, the 1- and 2-year metastasis-free survival rates were both 75% (33-100%). Among patients treated with sequential chemotherapy and 66 Gy of radiotherapy, the rates were 73% (±16%) and 66% (±20%) at 1 and 2 years, respectively. In patients concurrently treated with chemoradiotherapy, the metastasis-free survival was 73% (±16%) at both time-points. No statistically significant difference between the treatment groups was observed. Most commonly, distant metastases occurred simultaneously at multiple sites (5 patients) or separately in bone (8 patients), brain (5 patients) or liver (3 patients).

### Acute pulmonary and esophageal toxicity

Table2 summarizes the acute treatment-related pulmonary and esophageal toxicity of the patients. Of all patients, 13 (15%) experienced no acute pulmonary toxicity, 51 (60%) mild dry cough or dyspnea on exertion, and 20 (23%) grade 2 symptoms. Pulmonary toxicity did not statistically differ between treatment schedules. No acute esophageal toxicity was observed in 12 patients (13%), slight symptoms requiring no or non-narcotic analgesics in 43 (50%) and esophageal toxicity requiring narcotic analgesics and adapted diet in 30 patients (35%). Thereby, esophageal toxicity differed statistically significant between patients undergoing sequential or concurrent chemoradiotherapy, unfavorable for the latter (*p* = 0.013). Neither grade 3 nor grade 4 pulmonary or esophageal toxicity was observed and no patient died of treatment-related causes.

**Table 2:** Frequency (and % of patients in the respective treatment group) of acute pulmonary and esophageal toxicity in patients according to treatment

	Pulmonary toxicity			Esophageal toxicity		
Treatment	Grade 0	Grade 1	Grade 2	Grade 0	Grade 1	Grade 2
RT^$^	0 (0)	4 (57)	3 (43)	2 (29)	3 (43)	2 (29)
CHT-RT^#^	4 (10)	26 (62)	10 (24)	8 (19)	24 (57)	9 (21)
RCHT	9 (24)	21 (57)	7 (19)	2 (5)	16 (43)*	19 (51)*

^$^RT = radiotherapy alone; ^#^CHT-RT = sequential chemotherapy and radiotherapy; ^¶^RCHT = concurrent chemoradiotherapy. * *p* = 0.013; chi-square test of sequential *versus* concurrent chemoradiotherapy. For the sequential chemotherapy and radiotherapy group, numbers on acute pulmonary and esophageal toxicity are missing for 2 patients and 1 patient, respectively.

## Discussion

The number of clinical studies evaluating treatment outcome on the use of IMRT for locally advanced NSCLC is limited. The results reported in our study encourage the use of IMRT in patients with irresectable NSCLC. We found among all patients receiving 66 Gy 1- and 2-year overall survival rates of 71% and 56%, respectively, and a median overall survival of 29.7 months.

Several studies have addressed treatment outcome using 3D-CRT or IMRT for advanced stage NSCLC patients. Nakayama *et al*. compared the clinical results of high dose 3D-CRT (66 to 84 Gy) with those of conventional two-dimensional radiotherapy (56 to 66 Gy) for patients with Stage III NSCLC [13]. The overall survival rates at 3 years were 9.1% (95% CI, 0.7–18.9%) in the conventional group and 31.0% (95% CI, 18.9–43.1%) in the high-dose group. Wang *et al*. retrospectively evaluated the outcome of 237 stage III NSCLC patients treated with radiotherapy alone, sequential chemoradiotherapy or concurrent chemoradiotherapy using 3D-CRT [9]. The median overall survival of the entire cohort was 12.6 months, and 2- and 5-year overall survival rates were 22.4% and 10.0%, respectively. Recently, the Memorial Sloan-Kettering Cancer Center retrospectively reviewed treatment outcome of 55 stage I-IIIB inoperable NSCLC patients with large tumor volumes (GTV ≥ 100 cc) treated with IMRT [23]. For patients with stage III disease, 2-year local control and overall survival rates were both 58%, respectively, with a median survival time of 25 months. Liao *et al*. reviewed the records of 91 patients who received treatment with concurrent chemoradiotherapy using 4DCT/IMRT and compared outcome with patients treated with CT/3D-CRT [17]. The hazard ratio for 4DCT/IMRT was 0.64 (95% CI 0.41-0.98) and statistically significant (*p =* 0.039) for the overall survival. Based on these reports on 3D-CRT and IMRT [8,9,13,19], and our observations, we consider IMRT as an effective treatment option for patients with locally advanced NSCLC.

Besides favorable outcome data, no severe treatment-related acute pulmonary or esophageal toxicity was observed. In line with previous reports, however, acute esophageal toxicity was enhanced in concurrent schedules compared to sequential chemoradiotherapy [5,6]. Sura *et al*. scored acute toxicity on the RTOG scale and reported six patients (11%) to experience grade 3 acute pulmonary toxicity and two (4%) to experience grade 3 acute esophagitis, with no grade ≥4 acute toxicity [23]. Using the CTCAE version 3.0 scoring criteria [24], Yom *et al*. reported an incidence of grade ≥3 treatment-related pneumonitis in the IMRT group of 8% (95% CI, 4%–19%) at 6 and 12 months [16].

Differences in treatment outcome and toxicity may be attributable to different GTV volumes, delivered treatment schedules and radiation dose, tumor stage and the number of patients included in the study. In our study, the patient’s performance status was not explicitly taken into account. Nevertheless, the treatment choice is dependent on the performance score and only patients in good general condition, *i.e.*, Karnofsky index ≥70%, were considered candidates for concurrent chemoradiotherapy.

There are some concerns with respect to the use of IMRT in daily practice. One such concern is the effect of respiratory motion on the accuracy of IMRT delivery [25]. However, several studies have shown that dose variation, introduced by organ motion, almost completely disappears when delivering fractionated radiotherapy [26-28]. Furthermore, in our institute a slow-CT scan is routinely performed for treatment planning purposes incorporating all tumor motion into the definition of the GTV. By doing so, the respiratory motion has largely the same effect on IMRT dose distributions as on conformal radiotherapy techniques. Another concern of IMRT is low-dose radiation exposure of larger volumes of unaffected lung tissue. In the era of 3D-CRT, the mean lung dose (MLD; [29]) and the volume of unaffected lung tissue receiving a dose of at least 20 Gy (V20) were most often dose-limiting. By applying highly-conformal techniques such as IMRT, however, the MLD alone is more often dose limiting than V20. The reason for this is that by increasing the number of beams, *i.e.*, adding beams from various angles, a larger unaffected lung volume is exposed to a low dose of radiation (below 20 Gy, *i.e.*, V20 decreases). Furthermore, with conformal radiotherapy the PTV is always covered by all beams at all moments while an intrinsic feature of IMRT is that with IMRT, the entire PTV is not always covered by the beam in all its segments. This allows giving a higher dose to the PTV while V20 is unaffected, but with a higher MLD. That is explained by a somewhat higher total dose to the body as a result of increased monitor units delivered by more radiation beams [30,31]. As a consequence more radiation-induced cancers might be expected in long-term survivors [31,32].

Despite these concerns, IMRT outcome results are very promising and the toxicity rates acceptable, which supports the consideration of IMRT as an effective and useful radiotherapy technique for the treatment of locally advanced NSCLC.

Notwithstanding the encouraging results of IMRT already reported, more research is needed to further improve outcome for locally advanced NSCLC. To improve local control and outcome with modern radiotherapy techniques in stage III NSCLC patients, a modeling study, investigating the therapeutic gain of individualized dose prescription with dose escalation for various hypofractionation schemes, has recently been conducted at our institution [33]. The encouraging findings have resulted in a clinical phase II trial in which stage III NSCLC patients eligible for (concurrent chemo)radiotherapy are treated with individualized escalated dose.

## Conclusions

This retrospective cohort study in 86 patients with locally advanced irresectable non-small cell lung cancer shows that IMRT is an effective technique with acceptable acute toxicity, also when (sequentially or concomitantly) combined with chemotherapy.

## Competing interests

The authors declare to have no conflicts of interest.

## Authors’ contributions

SLAG data collection, analysis, preparation of manuscript. EGCT data collection, analysis, preparation of manuscript. OCJS preparation of manuscript. LFdeG-O preparation of manuscript. AT preparation of manuscript. PNS analysis, preparation of manuscript. JB analysis, preparation of manuscript. All authors read and approved the final manuscript.
